# Single-point insulin sensitivity estimator and left main and/or three-vessel disease in patients aged 45 years or older with acute coronary syndrome: findings from the CCC-ACS project

**DOI:** 10.3389/fendo.2026.1865655

**Published:** 2026-07-09

**Authors:** Mengchen Li, Yuyang Sun, Yu Liu, Yan Sun, Dai Zhang, Yujing Cheng, Xiaoli Liu

**Affiliations:** Department of Cardiology, Beijing Anzhen Hospital, Capital Medical University, Beijing Institute of Heart Lung and Blood Vessel Disease, Beijing, China

**Keywords:** acute coronary syndrome, coronary artery disease, high-risk coronary anatomy, insulin resistance, single-point insulin sensitivity estimator

## Abstract

**Background:**

Severe coronary anatomical involvement is clinically important in patients with acute coronary syndrome (ACS), yet evidence on its relationship with insulin sensitivity remains limited. This study examined the association of the single-point insulin sensitivity estimator (SPISE), a simple surrogate marker of insulin sensitivity, with severe coronary anatomical patterns, including left main and/or three-vessel disease and isolated three-vessel disease, in middle-aged and older patients with ACS.

**Methods:**

This cross-sectional analysis was based on data from the CCC-ACS project. A total of 16,383 patients were included in the analysis of left main and/or three-vessel disease, and 13,390 patients were included in the analysis of isolated three-vessel disease. SPISE was analyzed as both a continuous variable and according to tertiles. Multivariable logistic regression was used to evaluate the associations between SPISE and the study outcomes. Restricted cubic spline analyses were performed to assess dose-response patterns, and subgroup and sensitivity analyses were conducted to examine the robustness of the findings.

**Results:**

Higher SPISE was associated with lower odds of both severe coronary anatomical outcomes. In the fully adjusted model, each 1-unit increase in SPISE was associated with lower odds of left main and/or three-vessel disease (OR, 0.977; 95% CI, 0.957–0.998) and isolated three-vessel disease (OR, 0.957; 95% CI, 0.935–0.980). Compared with patients in the lowest SPISE tertile, those in the highest tertile had lower odds of both outcomes. Restricted cubic spline analyses supported dose-response relationships. Subgroup analyses suggested age-related heterogeneity, with the inverse associations mainly observed in patients aged <65 years and attenuated among those aged ≥65 years. Sensitivity analyses yielded generally consistent results.

**Conclusion:**

Among ACS patients aged 45 years or older, higher SPISE was associated with lower odds of severe coronary anatomical involvement, with this association mainly evident in patients aged <65 years. These findings support a potential link between reduced insulin sensitivity and angiographically defined severe coronary anatomical involvement and suggest that SPISE may serve as a readily available marker for identifying patients more likely to have high-risk coronary anatomy.

## 1 Introduction

With the progressive ageing of the population, cardiovascular disease (CVD) has become an increasingly important cause of illness and death worldwide, creating a substantial public health burden (1). Among the major clinical presentations of CVD, acute coronary syndrome (ACS) is a prominent contributor to death and disability (2). In patients with ACS, the extent of coronary anatomical involvement is closely linked to revascularization decision-making and clinical prognosis (3). Among these patients, left main and/or three-vessel disease generally indicates more extensive and severe coronary involvement and is associated with a greater ischemic burden, more complex treatment strategies, and poorer clinical outcomes. Although coronary angiography remains the standard method for evaluating such lesions, metabolic markers may offer additional value in recognizing severe coronary anatomical disease.

Insulin resistance (IR) has been implicated in both the onset and progression of atherosclerotic cardiovascular disease (4). Although the hyperinsulinemic-euglycemic clamp remains the reference method for evaluating IR, its complexity and high resource demands make it impractical for large epidemiologic studies and routine clinical use (5). As one of the conventional surrogate measures, homeostasis model assessment of insulin resistance (HOMA-IR) likewise depends on fasting insulin measurement and is constrained by issues related to assay availability, cost, and standardization (6). Beyond these traditional insulin-based indices, several non-insulin-derived surrogate markers have also been proposed. Among them, the triglyceride-glucose (TyG) index is commonly used because it can be derived readily from routine clinical data; however, its optimal cut-off values vary across populations, and its ability to capture obesity-related metabolic heterogeneity remains limited (7). The single-point insulin sensitivity estimator (SPISE) is a recently proposed non-insulin-based surrogate measure calculated from BMI, TG, and HDL-C, and may be used to approximate insulin sensitivity without direct insulin measurement (8). Recent studies indicate that SPISE is negatively related to IR and may show performance comparable to, or in some settings better than, HOMA-IR and other surrogate indices for detecting impaired insulin sensitivity and metabolic disturbances (9, 10). Nevertheless, current evidence has focused mainly on SPISE as a marker of IR, glucose metabolic disturbances, and metabolic syndrome (11, 12).

Although IR has been closely implicated in the progression of atherosclerosis, evidence regarding SPISE in middle-aged and older patients with ACS remains limited. In particular, direct evidence remains scarce for severe coronary anatomical patterns, including combined left main and multivessel involvement as well as isolated three-vessel involvement. Given that abnormalities in glucose and lipid metabolism are key manifestations of IR, and that glucose, total cholesterol, and low-density lipoprotein cholesterol (LDL-C) are all implicated in atherosclerotic progression, these metabolic factors may represent related metabolic context underlying the observed association between SPISE and severe coronary artery disease (13, 14). Therefore, in addition to examining the association between SPISE and severe coronary anatomical patterns, we performed exploratory mediation analyses to assess whether these related metabolic markers statistically accounted for part of the observed associations. Against this background, the present study used data from the Improving Care for Cardiovascular Disease in China–Acute Coronary Syndrome (CCC-ACS) project to investigate the association between SPISE and left main and/or three-vessel disease in middle-aged and older patients with ACS, and to further assess whether these related metabolic markers statistically accounted for part of the observed associations of related metabolic markers. In addition, to place the findings for SPISE in the context of established non-insulin-based IR indices, we performed supplementary comparative analyses using TyG and TyG-BMI.

## 2 Methods

### 2.1 Study design

The present analysis used data obtained from the CCC-ACS project, a national registry and quality improvement initiative for ACS care across China (15). The project was initiated in 2014 through a joint effort between the American Heart Association and the Chinese Society of Cardiology. Participating hospitals across mainland China consecutively enrolled eligible patients whose principal discharge diagnosis was ACS. Clinical information was entered into a web-based system and extracted from medical records by trained staff according to standardized definitions. To ensure data quality, real-time validation procedures, regular staff training, site audits, and third-party oversight were implemented. The CCC-ACS project was registered at ClinicalTrials.gov (NCT02306616). The study protocol received approval from the Ethics Committee of Beijing Anzhen Hospital, Capital Medical University (approval no. 2014018), and the requirement for informed consent was waived.

### 2.2 Study population

**Figure 1:**
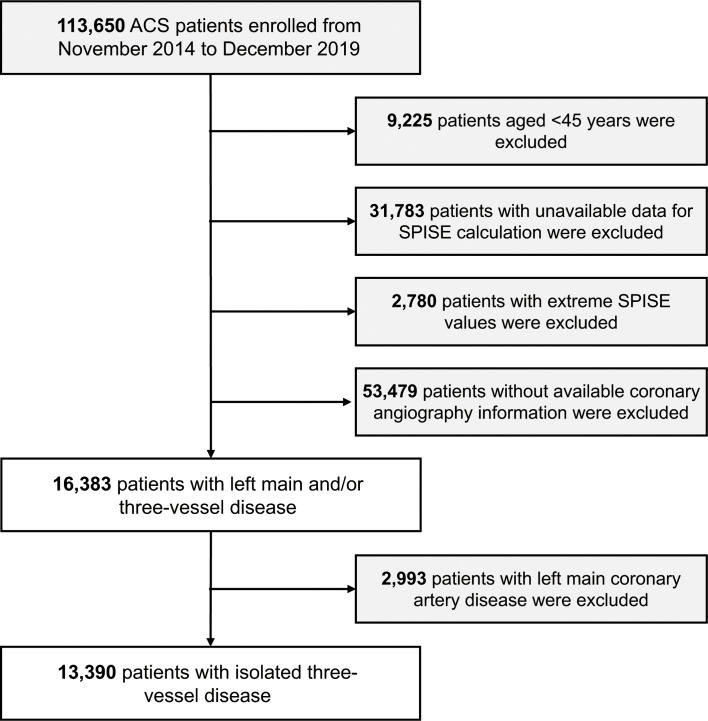
Flow chart of inclusion and exclusion criteria of participants. ACS, acute coronary syndrome; SPISE, Single-Point Insulin Sensitivity Estimator.

Between November 2014 and December 2019, the CCC-ACS project included 113,650 patients with ACS. Patients aged <45 years, those with unavailable data for SPISE calculation, those with extreme SPISE values, and those without available coronary angiography information were excluded. Finally, 16,383 patients were retained for the primary analysis of left main and/or three-vessel disease. For the secondary analysis of isolated three-vessel disease, 2,993 patients with left main coronary artery disease were further excluded, leaving 13,390 patients in the final study population. **Figure 1** illustrates the study population selection process. Detailed sequential exclusion counts, including component-specific missingness for SPISE calculation and vessel-specific missingness for coronary angiography information, are provided in **Supplementary Table 1**. The comparison between included and age-eligible excluded patients is shown in **Supplementary Table 2**. After exclusion of participants with missing required data, all variables included in the final multivariable models were complete.

### 2.3 Assessment of SPISE and supplementary IR indices

SPISE was derived using the equation developed by Paulmichl et al. (8): SPISE = 600 × HDL-C^0.185/(TG^0.2 × BMI^1.338), where HDL-C and TG were expressed in mg/dL and BMI in kg/m². Blood samples for lipid and glucose measurements were collected immediately after hospital admission as part of routine clinical care. TG and HDL-C values reported in mmol/L were converted to mg/dL before calculation in accordance with standard conversion factors. A lower SPISE value reflects poorer insulin sensitivity and greater IR. In the present analysis, SPISE was assessed both on a continuous scale and in tertiles determined from its distribution among the study participants.

For supplementary comparative analyses, we additionally calculated two established non-insulin-based IR indices, namely the triglyceride-glucose index and triglyceride-glucose-body mass index. The TyG index was calculated as ln [TG (mg/dL) × glucose (mg/dL)/2], and TyG-BMI was calculated as TyG × BMI. TG and glucose values reported in mmol/L were converted to mg/dL before calculation. Both TyG and TyG-BMI were analyzed as continuous variables and in tertiles using the same modeling strategy as that applied to SPISE.

### 2.4 Definitions of study outcomes

The primary outcome was left main and/or three-vessel disease identified on coronary angiography. A luminal diameter reduction of at least 50% in the left main coronary artery was considered left main disease. Three-vessel disease was considered present when all three major epicardial coronary arteries—the left anterior descending, left circumflex, and right coronary arteries—showed at least 50% diameter stenosis. The secondary outcome was isolated three-vessel disease, defined as three-vessel disease in the absence of left main disease. These outcomes were intended to capture the extent of major epicardial coronary artery involvement rather than detailed lesion morphology or functional ischemic significance. More comprehensive anatomical scoring systems, such as the SYNTAX or Gensini score, as well as physiological assessments were not available in the CCC-ACS database.

### 2.5 Assessment of covariates and candidate metabolic markers

On the basis of clinical importance and variable availability in the CCC-ACS database, covariates included age, sex, marital status, smoking status, history of hypertension, history of diabetes mellitus, prior myocardial infarction (MI), prior percutaneous coronary intervention (PCI), prior kidney failure, and ACS type. Marital status was categorized as married or others, with the latter including unmarried, divorced, widowed, and other marital status. Smoking within the past year was classified as current smoking. Histories of hypertension, diabetes mellitus, prior MI, and prior PCI were defined according to documented medical history before the index hospitalization. Prior kidney failure was defined as documented prior kidney failure or an admission estimated glomerular filtration rate (eGFR) <15 mL/min/1.73 m². ACS type was categorized as unstable angina pectoris, ST-segment elevation myocardial infarction, or non-ST-segment elevation myocardial infarction.

Glucose, total cholesterol, and LDL-C were assessed as candidate metabolic markers in exploratory mediation analyses. These variables were obtained from laboratory tests recorded at admission and were analyzed as continuous variables in the exploratory mediation analyses.

### 2.6 Statistical analyses

Baseline characteristics were summarized according to SPISE tertiles. Continuous variables are summarized as mean ± SD or median (interquartile range), as appropriate, whereas categorical variables are reported as counts and percentages. Across SPISE tertiles, continuous variables were compared by one-way analysis of variance or the Kruskal-Wallis test, and categorical variables by the chi-square test or Fisher’s exact test. Potential selection bias was assessed by comparing baseline covariates between patients included in the primary analysis and age-eligible excluded patients using standardized mean differences (SMDs), with SMD <0.10 indicating negligible imbalance.

The relationships of SPISE with left main and/or three-vessel disease and isolated three-vessel disease were assessed by logistic regression, with effect estimates expressed as ORs and 95% CIs. SPISE was modeled both continuously and in tertiles, with the first tertile serving as the reference. In the primary analyses, SPISE values outside the range bounded by the 1st and 99th percentiles were classified as extreme. Model 1 accounted for age and sex, Model 2 added marital status and smoking status, and Model 3 further incorporated history of hypertension, history of diabetes mellitus, prior MI, prior PCI, prior kidney failure, and ACS type. Trend tests were performed across SPISE tertiles.

Model diagnostics were performed to assess model stability. Multicollinearity was evaluated with the generalized variance inflation factor (GVIF), and no meaningful collinearity was observed among the covariates (**Supplementary Tables 3**, **4**). To address the comparison with other non-insulin-based IR indices, TyG and TyG-BMI were further evaluated in supplementary multivariable logistic regression analyses using the same covariate-adjustment strategy. The dose-response pattern and possible nonlinear association between SPISE and each outcome were examined using restricted cubic spline analyses based on multivariable logistic regression models. To enhance comparability with the tertile-based categorical analyses, 4 knots were placed at the 5th percentile, the two SPISE tertile cut-points, and the 95th percentile of the SPISE distribution. Odds ratios were estimated using the median SPISE value within the highest tertile as the reference, corresponding to an odds ratio of 1. The exact SPISE tertile cut-points, knot locations, and RCS reference values for each outcome are reported in **Supplementary Table 5**. The overall association and nonlinearity were assessed using Wald chi-square tests. The SPISE tertile cut-points were additionally indicated in the spline plots, and tertile-specific odds ratios from the categorical model were overlaid at the median SPISE value of each tertile.

Subgroup analyses were performed to evaluate whether the association between SPISE and the two coronary anatomical outcomes differed across clinically relevant strata, including age group (<65 and ≥65 years), sex, ACS type, history of diabetes mellitus, history of hypertension, marital status, smoking status, prior MI, prior PCI, and prior kidney failure. Within each subgroup, logistic regression models were adjusted for the same covariates as Model 3, except for the stratifying variable. Interaction was assessed by adding a cross-product term between continuous SPISE and the subgroup variable to the corresponding multivariable model. For ACS type, a global interaction test was used. Because multiple interaction tests were performed, false discovery rate correction was applied to the P values for interaction within each outcome. Subgroup analyses were considered exploratory, and both nominal and multiplicity-adjusted P values for interaction were reported.

Sensitivity and exploratory analyses were also performed. Three sensitivity analyses were conducted. In the first, the main analyses were repeated after including participants with extreme SPISE values. Second, we repeated the analyses after removing patients with cardiogenic shock or a history of cardiac arrest. Third, medication histories, including antihypertensive, antidiabetic, and lipid-lowering medication use, were additionally adjusted for on the basis of Model 3. As an exploratory analysis, we performed mediation analyses to examine whether glucose, total cholesterol, and LDL-C statistically accounted for part of the associations between SPISE and the two coronary anatomical outcomes. Candidate mediators were examined one at a time, and the average causal mediation effect, average direct effect, total effect, and mediated proportion were reported with 95% confidence intervals. The results were interpreted cautiously as statistical decomposition of the observed associations.

R software (version 4.5.2) was used for all statistical analyses. Statistical significance was defined as a two-sided P value of less than 0.05.

## 3 Results

### 3.1 Baseline characteristics of the study population

In the cross-sectional analyses, the primary outcome of left main and/or three-vessel disease was assessed in 16,383 ACS patients, while the secondary outcome of isolated three-vessel disease was assessed in 13,390. Compared with age-eligible patients excluded from the primary analysis, included patients were broadly comparable for most baseline covariates. Baseline characteristics by SPISE tertiles are presented in **Supplementary Tables 6**, **7**. Across both analyses, higher SPISE tertiles were characterized by older age, lower proportions of men and smokers, lower prevalence of hypertension, and fewer histories of prior MI and prior PCI, while marital status and prior kidney failure were broadly similar among tertiles. Diabetes history decreased across higher SPISE tertiles only in the primary analysis. With increasing SPISE tertiles, BMI, TC, TG, LDL-C, and glucose levels showed a decreasing trend, whereas HDL-C increased progressively (all P < 0.01).

### 3.2 Association between SPISE and left main and/or three-vessel disease

**Figure 2:**
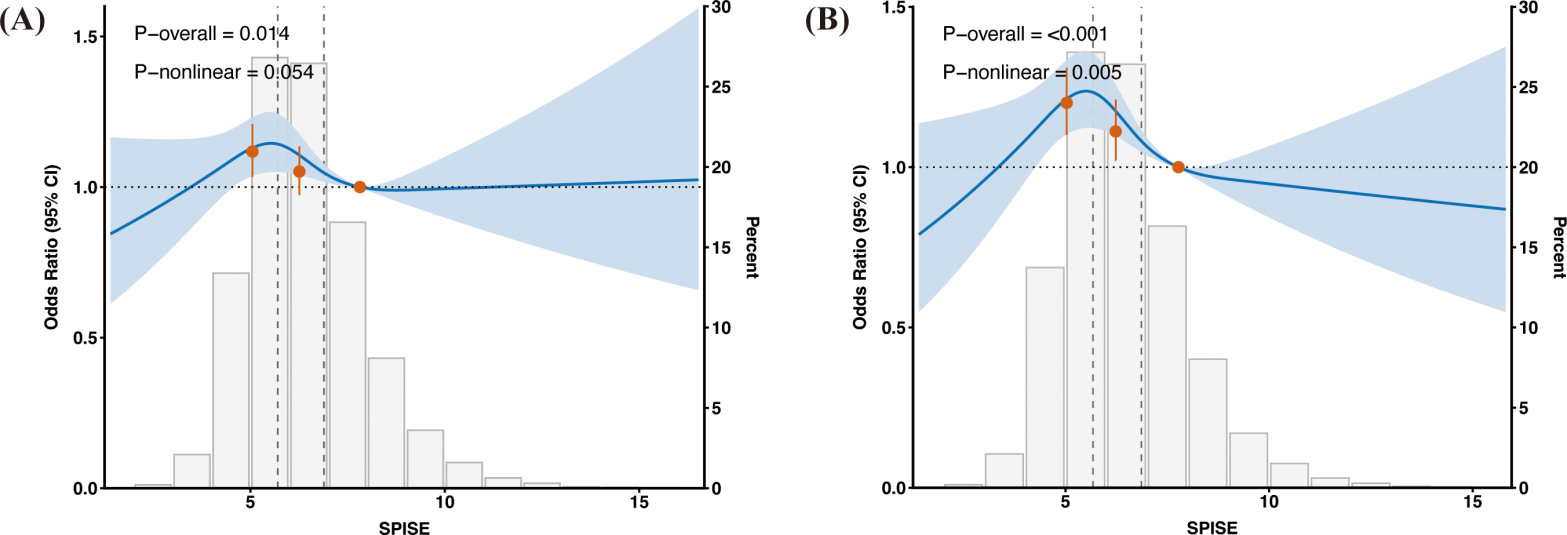
Multivariable-adjusted restricted cubic spline analysis of SPISE in relation to left main and/or three-vessel disease **(A)** and isolated three-vessel disease **(B)**. To improve comparability with tertile-based analyses, four knots were placed at the 5th percentile, the two SPISE tertile cut-points, and the 95th percentile; exact knot locations and reference values are provided in **Supplementary Table 5**. Vertical dashed lines indicate tertile cut-points, and orange points represent tertile-specific ORs at the median SPISE value of each tertile. Models were adjusted for age, sex, ACS type, history of diabetes mellitus, history of hypertension, marital status, smoking status, prior MI, prior PCI, and prior kidney failure. SPISE, single-point insulin sensitivity estimator; OR, odds ratio; CI, confidence interval; ACS, acute coronary syndrome; MI, myocardial infarction; PCI, percutaneous coronary intervention.

**Table 1** shows the cross-sectional associations of SPISE with left main and/or three-vessel disease. Across all models, higher SPISE consistently corresponded to lower odds of high-risk coronary anatomy. In Model 3, after accounting for age, sex, marital status, smoking status, history of hypertension, history of diabetes mellitus, prior MI, prior PCI, prior kidney failure, and ACS type, each 1-unit increase in SPISE corresponded to a 2.3% reduction in the odds of high-risk coronary anatomy (OR, 0.977; 95% CI, 0.957–0.998; P = 0.028). In tertile-based analyses, participants in the top SPISE tertile had significantly reduced odds of left main and/or three-vessel disease compared with those in the bottom tertile (OR, 0.895; 95% CI, 0.827–0.968; P = 0.005). SPISE tertiles also showed a significant dose-response relationship (P for trend = 0.006). In the multivariable-adjusted RCS model (**Figure 2A**), SPISE was significantly associated with left main and/or three-vessel disease overall (P-overall = 0.014), whereas the evidence for nonlinearity was borderline (P for nonlinearity = 0.054).

**Table 1:** Association of SPISE with left main and/or three-vessel disease.

Exposure	Model 1		Model 2		Model 3	
	OR (95% CI)	P value	OR (95% CI)	P value	OR (95% CI)	P value
SPISE index, per 1-unit increase	0.973 (0.953, 0.993)	0.009	0.973 (0.953, 0.993)	0.009	0.977 (0.957, 0.998)	0.028
SPISE index, tertiles						
Tertile 1	Reference		Reference		Reference	
Tertile 2	0.935 (0.866, 1.009)	0.084	0.934 (0.866, 1.008)	0.080	0.941 (0.871, 1.016)	0.119
Tertile 3	0.883 (0.817, 0.955)	0.002	0.883 (0.817, 0.954)	0.002	0.895 (0.827, 0.968)	0.005
P for trend		0.002		0.002		0.006

SPISE, single-point insulin sensitivity estimator; OR, odds ratio; CI, confidence interval; MI, myocardial infarction; PCI, percutaneous coronary intervention.

Model 1: adjusted for age, sex.

Model 2: adjusted for age, sex, marital status, and smoking status.

Model 3: adjusted for age, sex, marital status, smoking status, history of hypertension, history of diabetes mellitus, prior MI, prior PCI, prior kidney failure, and ACS type.

In supplementary comparative analyses, higher TyG and higher TyG-BMI tertiles were associated with increased odds of left main and/or three-vessel disease, directionally consistent with the inverse association observed for SPISE (**Supplementary Table 8**).

### 3.3 Association between SPISE and isolated three-vessel disease

**Table 2** summarizes the cross-sectional associations between SPISE and isolated three-vessel disease. Across all models, higher SPISE consistently corresponded to lower odds of isolated three-vessel disease. In Model 3, after accounting for age, sex, marital status, smoking status, history of hypertension, history of diabetes mellitus, prior MI, prior PCI, prior kidney failure, and ACS type, each 1-unit increase in SPISE corresponded to a 4.3% reduction in the odds of isolated three-vessel disease (OR, 0.957; 95% CI, 0.935–0.980; P < 0.001). In tertile-based analyses, compared with participants in the lowest SPISE tertile, those in the highest tertile had significantly lower odds of isolated three-vessel disease (OR, 0.833; 95% CI, 0.763–0.909; P < 0.001). There was also clear evidence of a dose-response pattern across SPISE tertiles (P for trend < 0.001). In the multivariable-adjusted RCS model (**Figure 2B**), SPISE was significantly associated with isolated three-vessel disease, with significant evidence of nonlinearity (overall P < 0.001; P for nonlinearity = 0.005). The spline curve suggested a nonlinear pattern, with higher odds observed in the lower-to-middle range of SPISE, followed by a decline toward higher SPISE levels.

**Table 2:** Association of SPISE with isolated three-vessel disease.

Exposure	Model 1		Model 2		Model 3	
	OR (95% CI)	P value	OR (95% CI)	P value	OR (95% CI)	P value
SPISE index, per 1-unit increase	0.954 (0.932, 0.976)	<0.001	0.954 (0.932, 0.976)	<0.001	0.957 (0.935, 0.980)	<0.001
SPISE index, tertiles						
Tertile 1	Reference		Reference		Reference	
Tertile 2	0.914 (0.840, 0.994)	0.036	0.913 (0.839, 0.993)	0.034	0.926 (0.850, 1.008)	0.076
Tertile 3	0.826 (0.758, 0.900)	<0.001	0.826 (0.758, 0.900)	<0.001	0.833 (0.763, 0.909)	<0.001
P for trend		<0.001		<0.001		<0.001

SPISE, single-point insulin sensitivity estimator; OR, odds ratio; CI, confidence interval; MI, myocardial infarction; PCI, percutaneous coronary intervention.

Model 1: adjusted for age, sex.

Model 2: adjusted for age, sex, marital status, and smoking status.

Model 3: adjusted for age, sex, marital status, smoking status, history of hypertension, history of diabetes mellitus, prior MI, prior PCI, prior kidney failure, and ACS type.

In supplementary comparative analyses, higher TyG and TyG-BMI were both associated with increased odds of isolated three-vessel disease. These results were directionally consistent with the inverse association observed for SPISE and further supported the relevance of IR-related metabolic status to severe coronary anatomical involvement (**Supplementary Table 9**).

### 3.4 Subgroup analyses

**Figure 3:**
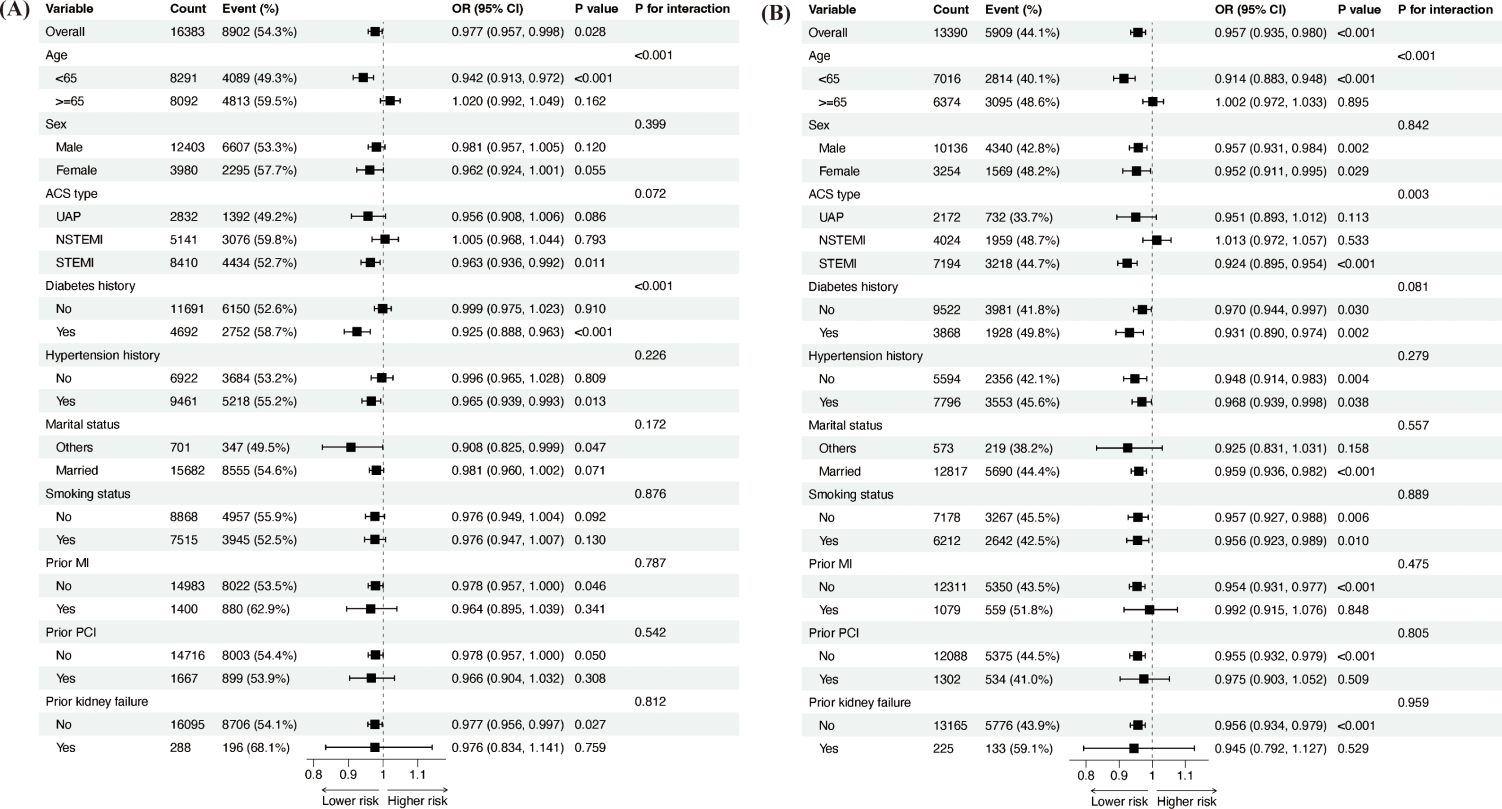
Subgroup and interaction analyses of the association between SPISE and left main and/or three-vessel disease **(A)** and isolated three-vessel disease **(B)**. Adjusted for age, sex, marital status, smoking status, hypertension history, diabetes history, ACS type, prior MI, prior PCI, and prior kidney failure. SPISE, single-point insulin sensitivity estimator; MI, myocardial infarction; PCI, percutaneous coronary intervention; OR, odds ratio; CI, confidence interval; ACS, acute coronary syndrome.

**Figure 3** shows subgroup analyses for left main and/or three-vessel disease and isolated three-vessel disease. For left main and/or three-vessel disease (**Figure 3A**), age showed significant effect modification (P for interaction < 0.001); the inverse association between SPISE and the outcome was evident among patients aged <65 years but was attenuated and statistically non-significant among those aged ≥65 years. Diabetes history also showed evidence of interaction, whereas no robust interactions were observed for the other subgroup variables. For isolated three-vessel disease (**Figure 3B**), a similar age-related pattern was observed, with a significant age interaction (P for interaction < 0.001) and an inverse association confined mainly to patients aged <65 years. ACS type also showed evidence of interaction, while the remaining subgroup variables did not show robust interactions. The corresponding FDR-adjusted and Bonferroni-adjusted P values for interaction are presented in **Supplementary Table 10**.

### 3.5 Sensitivity and exploratory analyses

Results from the sensitivity analyses were in line with those of the primary analyses. The first sensitivity analysis showed that keeping participants with extreme SPISE values did not substantially affect the associations of SPISE with the two coronary anatomical outcomes. Higher SPISE remained associated with lower odds of both outcomes, and the dose-response trends across SPISE tertiles persisted (**Supplementary Tables 11**, **12**). Results remained largely unchanged in the second sensitivity analysis after patients with cardiogenic shock or cardiac arrest were excluded. Higher SPISE continued to be associated with lower odds of left main and/or three-vessel disease and isolated three-vessel disease, with significant trends across SPISE tertiles remaining evident (**Supplementary Tables 13**, **14**). Additionally, further adjustment for medication histories for diabetes mellitus, hypertension, and dyslipidemia on the basis of Model 3 yielded generally consistent results. Higher SPISE remained inversely associated with the odds of both coronary anatomical outcomes, and the overall trend across SPISE tertiles was preserved (**Supplementary Tables 15**, **16**). These findings support the robustness of the main results.

Exploratory mediation analyses are presented in **Supplementary Table 17**. For both left main and/or three-vessel disease and isolated three-vessel disease, glucose, total cholesterol, and LDL-C showed statistically significant indirect effects in the associations with SPISE. Among these markers, glucose showed the largest mediated proportion. However, given the cross-sectional design and the close biological relationships between SPISE and cardiometabolic markers, these findings should be interpreted as exploratory statistical decomposition rather than evidence of causal mediation.

## 4 Discussion

This study examined the relationship of SPISE to severe coronary anatomical involvement among middle-aged and older patients with ACS. Across both continuous and tertile-based analyses, lower SPISE levels consistently indicated a higher probability of left main and/or three-vessel disease and isolated three-vessel disease. Restricted cubic spline analyses further suggested a generally linear inverse association of SPISE with left main and/or three-vessel disease, whereas a degree of nonlinearity was observed in the association with isolated three-vessel disease. Subgroup and sensitivity analyses generally supported the robustness of the main findings. In addition, exploratory mediation analyses suggested that related metabolic markers, particularly glucose, may statistically account for part of the observed associations.

The present study showed that reduced SPISE was significantly associated with increased odds of both severe coronary anatomical phenotypes, which is broadly consistent with prior evidence regarding other surrogate measures of IR. Earlier evidence suggests that an elevated TyG index is related to a higher likelihood of multivessel coronary artery disease in ACS (16). Cheng et al. also reported a positive relationship between TG/HDL-C and coronary artery disease severity (17). Recent evidence has likewise shown a significant positive association between triglyceride-glucose-body mass index (TyG-BMI) and multivessel coronary disease (18). In line with these previous findings, our supplementary comparative analyses showed that higher TyG and TyG-BMI were positively associated with left main and/or three-vessel disease and isolated three-vessel disease. These findings were directionally consistent with the inverse associations observed for SPISE, further supporting the relevance of IR-related metabolic status to severe coronary anatomical involvement. Compared with other surrogate indices, previous studies on SPISE have focused more on incident CVD or other adverse cardiovascular outcomes, with relatively limited attention to coronary anatomical severity itself (19–21). In this context, our study extends the evidence base for the potential application of SPISE in identifying the risk of severe coronary artery disease. In addition, RCS analysis further supported an overall association between SPISE and left main and/or three-vessel disease, with only borderline evidence of nonlinearity. In contrast, the association of SPISE with isolated three-vessel disease was significantly nonlinear, suggesting that its relationship with different severe coronary anatomical phenotypes may not be entirely uniform. These findings are broadly consistent with earlier reports of a negative association between SPISE and adverse cardiovascular outcomes, while also suggesting that the dose-response relationship may differ according to the specific cardiovascular phenotype under investigation (20, 21).

Analyses across subgroups suggested that the association between SPISE and severe coronary anatomical involvement was not completely uniform across clinical strata. Notably, age significantly modified this association. For both left main and/or three-vessel disease and isolated three-vessel disease, the inverse association was more evident in younger patients and became less pronounced among individuals aged 65 years or above. In younger individuals, glucose and lipid metabolic disturbances related to IR may have a stronger influence on extensive coronary involvement (22), whereas in older patients, a more complex clinical background may partly weaken the association between SPISE and coronary anatomical phenotypes (23). In the exploratory mediation analyses, glucose, total cholesterol, and LDL-C statistically accounted for part of the associations between SPISE and the two coronary anatomical outcomes, with glucose showing the largest estimated proportion. Nevertheless, given the cross-sectional nature of the data and the shared cardiometabolic context of these markers, these findings should be interpreted cautiously as exploratory statistical findings rather than evidence of causal mediation. This interpretation is also consistent with previous evidence highlighting the complex interplay between insulin resistance and dyslipidemia (24).

Although these exploratory findings suggest that glucose and lipid markers may help characterize the metabolic context of the association between SPISE and severe coronary anatomical involvement, this relationship is likely to reflect a broader and more complex cardiometabolic phenotype rather than a single explanatory pathway. Previous research suggests that, beyond its links with glucose and lipid metabolic disturbances, IR may contribute to the development and progression of atherosclerotic plaques via endothelial dysfunction, oxidative stress, chronic low-grade inflammatory activity, and thrombosis-related alterations (25, 26). As a validated surrogate index for IR, SPISE has shown good ability to detect impaired insulin sensitivity, IR, and metabolic syndrome (11). A lower SPISE may therefore reflect a less favorable overall cardiometabolic profile and, to some extent, capture the atherosclerotic burden associated with hypertriglyceridemia, low HDL-C, and adiposity-related metabolic dysregulation. Existing evidence suggests that hypertriglyceridemia and reduced HDL-C are typical features of lipid dysregulation in insulin resistance and are linked to higher atherosclerotic risk (24, 27). At the same time, the excess adiposity and metabolic burden reflected by BMI have also been consistently linked to IR and unfavorable cardiovascular phenotypes (28, 29). Taken together, SPISE may serve not only as a simple indicator of insulin sensitivity, but also as a potentially useful metabolic marker for recognizing severe coronary anatomical involvement in middle-aged and older patients with ACS.

One important strength of the study is that it drew on data from the CCC-ACS project, a nationwide multicenter real-world registry, and specifically examined aged 45 years and older patients with ACS. Using left main and/or three-vessel disease and isolated three-vessel disease as two clinically meaningful phenotypes of severe coronary anatomical involvement, we systematically evaluated the association between SPISE and severe coronary disease burden. Because SPISE can be derived inexpensively from routine clinical data, the present findings suggest that it may serve as a simple metabolic marker associated with concomitant severe coronary anatomical involvement among individuals with ACS.

Several limitations of this study should also be acknowledged. First, the observational cross-sectional design precludes causal inference and does not completely rule out residual confounding. Accordingly, temporal ordering among SPISE, candidate metabolic markers, and coronary anatomical outcomes could not be established, and the exploratory mediation analyses should be interpreted as hypothesis-generating rather than evidence of causal mediation. Second, SPISE was calculated from a single set of measurements obtained immediately after hospital admission. Because metabolic indicators may be influenced by fasting status, early treatment, and acute stress responses in ACS, admission-based SPISE may not fully reflect long-term insulin sensitivity. Future studies with standardized or repeated measurements are warranted. Third, severe coronary disease was defined by angiographic involvement of the left main and/or three major coronary arteries. This clinically meaningful definition captures the extent of coronary involvement but does not reflect detailed lesion complexity or functional significance. Because SYNTAX/Gensini scores and physiological assessments were unavailable in the CCC-ACS project, our findings should be interpreted as associations with angiographically defined severe anatomical involvement rather than overall lesion complexity or ischemic burden. Fourth, the study population was restricted to patients aged ≥45 years. Therefore, the findings may not be generalizable to younger patients with premature ACS, in whom metabolic dysfunction and insulin resistance may have distinct clinical implications. Future studies including younger ACS populations are warranted to determine whether the association between SPISE and severe coronary anatomical involvement differs by age. In addition, although medication histories were additionally adjusted for in sensitivity analyses, detailed information on medication dose, duration, adherence, and in-hospital treatment was unavailable, and residual confounding related to medication use cannot be completely excluded. Finally, the exclusion of patients with missing SPISE components or incomplete coronary angiography information may have introduced selection bias, although most measured baseline covariates were generally balanced between included and excluded age-eligible patients.

## 5 Conclusions

In this study of ACS patients aged 45 years or older, higher SPISE was associated with lower odds of left main and/or three-vessel disease and isolated three-vessel disease. However, subgroup analyses suggested that this inverse association was mainly evident among patients aged <65 years and was attenuated among those aged ≥65 years. SPISE may help identify ACS patients more likely to have severe coronary anatomical involvement, particularly in non-elderly patients, whereas its utility in elderly patients requires further validation.

## Data Availability

The dataset contains patient-level clinical information and is not publicly available due to privacy and ethical restrictions. It may be available from the corresponding author upon reasonable request and with appropriate approval. Requests to access these datasets should be directed to Xiaoli Liu, liuxiaoli@ccmu.edu.cn.
