# Supplementary material for: Single-point insulin sensitivity estimator and left main and/or three-vessel disease in patients aged 45 years or older with acute coronary syndrome: findings from the CCC-ACS project
Source: Front Endocrinol (Lausanne). 2026 Jul 9;17:1865655. doi: 10.3389/fendo.2026.1865655 (PMC13391384; doi:10.3389/fendo.2026.1865655)
Supplement: Supplementary file 1 [file Table1.docx]

Supplementary Material

**Supplementary Table 1.** Detailed exclusion process for the analytic cohort.

| **Exclusion Step** | **Excluded (n)** | **Remaining (n)** |
| --- | --- | --- |
| **1. Initial ACS patients** | - | 113,650 |
| **2. Excluded Age <45 years** | 9,225 | 104,425 |
| **3. Excluded Missing data for SPISE calculation** |  |  |
| 3.1 Excluded missing BMI data | 31,783 | 72,642 |
| 3.2 Excluded missing triglyceride data | 0 | 72,642 |
| 3.3 Excluded missing HDL-C data | 0 | 72,642 |
| **4. Excluded Extreme SPISE values** | 2,780 | 69,862 |
| **5. Excluded Missing coronary angiography information** | |  |
| 5.1 Excluded missing left main coronary artery information | 51,394 | 18,468 |
| 5.2 Excluded missing left anterior descending artery information | 848 | 17,620 |
| 5.3 Excluded missing left circumflex artery information | 816 | 16,804 |
| 5.4 Excluded missing right coronary artery information | 421 | 16,383 |
| **6. Final primary analysis cohort** | - | 16,383 |
| **7. Left main coronary artery disease excluded** | 2,993 | 13,390 |
| **8. Final secondary analysis cohort** | - | 13,390 |

**Supplementary Table 2.** Standardized mean difference comparison between included and excluded age-eligible patients.

| **Variable** | **SMD** |
| --- | --- |
| Age | 0.053 |
| Sex | 0.104 |
| Marital status | 0.057 |
| Smoking status | 0.107 |
| Prior MI | 0.01 |
| Prior PCI | 0.043 |
| Prior kidney failure | 0.003 |
| History of hypertension | 0.069 |
| History of diabetes mellitus | 0.13 |
| ACS type | 0.212 |

SMD, Standardized mean difference; MI, myocardial infarction; PCI, percutaneous coronary intervention; ACS, acute coronary syndrome.

**Supplementary Table 3.** Multicollinearity diagnostics for covariates in the cross-sectional analysis of left main and/or three-vessel disease.

| Characteristics | GVIF | DF | GVIF^(1/(2*Df)) |
| --- | --- | --- | --- |
| SPISE | 1.077 | 1 | 1.038 |
| Age | 1.166 | 1 | 1.08 |
| Sex | 1.268 | 1 | 1.126 |
| Marital status | 1.007 | 1 | 1.004 |
| Smoking status | 1.259 | 1 | 1.122 |
| History of hypertension | 1.064 | 1 | 1.032 |
| History of diabetes mellitus | 1.042 | 1 | 1.021 |
| Prior MI | 1.522 | 1 | 1.234 |
| Prior PCI | 1.561 | 1 | 1.249 |
| Prior kidney failure | 1.016 | 1 | 1.008 |
| ACS type | 1.052 | 2 | 1.013 |

SPISE, single-point insulin sensitivity estimator; GVIF, generalized variance inflation factor; DF, degrees of freedom; MI, myocardial infarction; PCI, percutaneous coronary intervention; ACS, acute coronary syndrome.

**Supplementary Table 4.** Multicollinearity diagnostics for covariates in the cross-sectional analysis of isolated three-vessel disease.

| Characteristics | GVIF | DF | GVIF^(1/(2*Df)) |
| --- | --- | --- | --- |
| SPISE | 1.073 | 1 | 1.036 |
| Age | 1.17 | 1 | 1.082 |
| Sex | 1.278 | 1 | 1.131 |
| Marital status | 1.007 | 1 | 1.004 |
| Smoking status | 1.271 | 1 | 1.127 |
| History of hypertension | 1.062 | 1 | 1.03 |
| History of diabetes mellitus | 1.042 | 1 | 1.021 |
| Prior MI | 1.604 | 1 | 1.267 |
| Prior PCI | 1.636 | 1 | 1.279 |
| Prior kidney failure | 1.016 | 1 | 1.008 |
| ACS type | 1.049 | 2 | 1.012 |

SPISE, single-point insulin sensitivity estimator; GVIF, generalized variance inflation factor; DF, degrees of freedom; MI, myocardial infarction; PCI, percutaneous coronary intervention; ACS, acute coronary syndrome.

**Supplementary Table 5.** Restricted cubic spline knot locations, reference values, and P values for nonlinear associations.

| **Outcome** | **Knot 1** | **Knot 2** | **Knot 3** | **Knot 4** | **Reference value** | **P-overall** | **P-nonlinearity** |
| --- | --- | --- | --- | --- | --- | --- | --- |
| Left main and/or three-vessel disease | 4.317 | 5.702 | 6.891 | 9.312 | 7.816 | 0.014 | 0.054 |
| Isolated three-vessel disease | 4.311 | 5.676 | 6.864 | 9.240 | 7.774 | <0.001 | 0.005 |

Knots were placed at the 5th percentile, the two SPISE tertile cut-points, and the 95th percentile of the SPISE distribution to improve comparability with tertile-based analyses. The reference value was defined as the median SPISE value within the highest tertile. Models were adjusted for age, sex, ACS type, history of diabetes mellitus, history of hypertension, marital status, smoking status, prior MI, prior PCI, and prior kidney failure. SPISE, single-point insulin sensitivity estimator; ACS, acute coronary syndrome; MI, myocardial infarction; PCI, percutaneous coronary intervention.

**Supplementary Table 6.** Baseline characteristics of participants across SPISE groups in the cross-sectional analysis of left main and/or three-vessel disease.

| **Characteristic** | **Overall  (N=16383)** | **SPISE** | | | ***P* value** |
| --- | --- | --- | --- | --- | --- |
|  |  | **T1 (N=5461)** | **T2 (N=5461)** | **T3 (N=5461)** |  |
| **Age, years** | 64.00 [57.00, 72.00] | 62.00 [54.00, 69.00] | 64.00 [57.00, 72.00] | 67.00 [60.00, 75.00] | <0.01 |
| **Sex** |  |  |  |  | <0.01 |
| Male | 12403 (75.71) | 4284 (78.45) | 4178 (76.51) | 3941 (72.17) |  |
| Female | 3980 (24.29) | 1177 (21.55) | 1283 (23.49) | 1520 (27.83) |  |
| **Marital status** |  |  |  |  | 0.41 |
| Married | 15682 (95.72) | 5235 (95.86) | 5236 (95.88) | 5211 (95.42) |  |
| Others | 701 (4.28) | 226 (4.14) | 225 (4.12) | 250 (4.58) |  |
| **Smoking status** |  |  |  |  | <0.01 |
| No | 8868 (54.13) | 2785 (51.00) | 2959 (54.18) | 3124 (57.21) |  |
| Yes | 7515 (45.87) | 2676 (49.00) | 2502 (45.82) | 2337 (42.79) |  |
| **History of hypertension** | |  |  |  |  |
| No | 6922 (42.25) | 2163 (39.61) | 2334 (42.74) | 2425 (44.41) | <0.01 |
| Yes | 9461 (57.75) | 3298 (60.39) | 3127 (57.26) | 3036 (55.59) |  |
| **History of diabetes mellitus** | |  |  |  | <0.01 |
| No | 11691 (71.36) | 3813 (69.82) | 3926 (71.89) | 3952 (72.37) |  |
| Yes | 4692 (28.64) | 1648 (30.18) | 1535 (28.11) | 1509 (27.63) |  |
| **Prior MI** |  |  |  |  | <0.01 |
| No | 14983 (91.45) | 4938 (90.42) | 4999 (91.54) | 5046 (92.40) |  |
| Yes | 1400 (8.55) | 523 (9.58) | 462 (8.46) | 415 (7.60) |  |
| **Prior PCI** |  |  |  |  | <0.01 |
| No | 14716 (89.82) | 4831 (88.46) | 4932 (90.31) | 4953 (90.70) |  |
| Yes | 1667 (10.18) | 630 (11.54) | 529 (9.69) | 508 (9.30) |  |
| **Prior kidney failure** |  |  |  |  | 0.16 |
| No | 16095 (98.24) | 5355 (98.06) | 5380 (98.52) | 5360 (98.15) |  |
| Yes | 288 (1.76) | 106 (1.94) | 81 (1.48) | 101 (1.85) |  |
| **ACS type** |  |  |  |  | <0.01 |
| UAP | 2832 (17.29) | 939 (17.19) | 992 (18.17) | 901 (16.50) |  |
| STEMI | 8410 (51.33) | 2785 (51.00) | 2721 (49.83) | 2904 (53.18) |  |
| NSTEMI | 5141 (31.38) | 1737 (31.81) | 1748 (32.01) | 1656 (30.32) |  |
| **BMI, kg/m²** | 24.22 [22.31, 26.12] | 26.99 [25.39, 28.67] | 24.22 [23.14, 25.39] | 21.64 [20.21, 22.99] | <0.01 |
| **Total cholesterol, mg/dL** | 168.60 [140.37, 198.76] | 172.08 [142.31, 203.02] | 168.21 [139.99, 197.99] | 165.26 [138.83, 195.08] | <0.01 |
| **Triglycerides, mg/dL** | 129.31 [93.00, 188.65] | 190.43 [138.17, 265.71] | 129.31 [100.97, 171.83] | 91.23 [70.86, 119.57] | <0.01 |
| **HDL-C, mg/dL** | 39.44 [33.64, 47.18] | 35.58 [30.55, 41.38] | 39.06 [34.03, 46.02] | 44.47 [37.90, 52.98] | <0.01 |
| **LDL-C, mg/dL** | 104.02 [80.82, 129.16] | 106.34 [82.75, 130.70] | 105.18 [82.21, 130.70] | 100.16 [78.11, 126.06] | <0.01 |
| **Glucose, mmol/L** | 6.06 [5.10, 7.84] | 6.39 [5.28, 8.27] | 6.01 [5.09, 7.86] | 5.82 [4.94, 7.50] | <0.01 |
| **High-risk coronary**  **anatomy, n (%)** |  |  |  |  | 0.97 |
| No | 7481 (45.66) | 2491 (45.61) | 2501 (45.80) | 2489 (45.58) |  |
| Yes | 8902 (54.34) | 2970 (54.39) | 2960 (54.20) | 2972 (54.42) |  |

SPISE tertiles were defined as T1, 1.40 to <5.70; T2, 5.70 to <6.89; and T3, 6.89 to 16.52. Normally distributed continuous variables are shown as mean ± SD, non-normally distributed continuous variables as median (IQR), and categorical variables as n (%). SPISE, single-point insulin sensitivity estimator; T1, tertile 1; T2, tertile 2; T3, tertile 3; N, number; BMI, body mass index; HDL-C, high-density lipoprotein cholesterol; LDL-C, low-density lipoprotein cholesterol; MI, myocardial infarction; PCI, percutaneous coronary intervention; ACS, acute coronary syndrome; UAP, unstable angina pectoris; STEMI, ST-segment elevation myocardial infarction; NSTEMI, non-ST-segment elevation myocardial infarction.

**Supplementary Table 7.** Baseline characteristics of participants across SPISE groups in the cross-sectional analysis of isolated three-vessel disease.

| **Characteristic** | **Overall  (N=13390)** | **SPISE** | | | ***P* value** |
| --- | --- | --- | --- | --- | --- |
|  |  | **T1 (N=4464)** | **T2 (N=4463)** | **T3 (N=4463)** |  |
| **Age, years** | 64.00 [56.00, 71.00] | 61.00 [54.00, 68.00] | 64.00 [57.00, 71.00] | 67.00 [60.00, 74.00] | <0.01 |
| **Sex** |  |  |  |  | <0.01 |
| Male | 10136 (75.70) | 3507 (78.56) | 3434 (76.94) | 3195 (71.59) |  |
| Female | 3254 (24.30) | 957 (21.44) | 1029 (23.06) | 1268 (28.41) |  |
| **Marital status** |  |  |  |  | 0.16 |
| Married | 12817 (95.72) | 4281 (95.90) | 4285 (96.01) | 4251 (95.25) |  |
| Others | 573 (4.28) | 183 (4.10) | 178 (3.99) | 212 (4.75) |  |
| **Smoking status** |  |  |  |  | <0.01 |
| No | 7178 (53.61) | 2255 (50.52) | 2388 (53.51) | 2535 (56.80) |  |
| Yes | 6212 (46.39) | 2209 (49.48) | 2075 (46.49) | 1928 (43.20) |  |
| **History of hypertension** | |  |  |  | <0.01 |
| No | 5594 (41.78) | 1778 (39.83) | 1912 (42.84) | 1904 (42.66) |  |
| Yes | 7796 (58.22) | 2686 (60.17) | 2551 (57.16) | 2559 (57.34) |  |
| **History of diabetes mellitus** | |  |  |  | 0.24 |
| No | 9522 (71.11) | 3139 (70.32) | 3211 (71.95) | 3172 (71.07) |  |
| Yes | 3868 (28.89) | 1325 (29.68) | 1252 (28.05) | 1291 (28.93) |  |
| **Prior MI** |  |  |  |  | 0.03 |
| No | 12311 (91.94) | 4068 (91.13) | 4108 (92.05) | 4135 (92.65) |  |
| Yes | 1079 (8.06) | 396 (8.87) | 355 (7.95) | 328 (7.35) |  |
| **Prior PCI** |  |  |  |  | 0.02 |
| No | 12088 (90.28) | 3986 (89.29) | 4041 (90.54) | 4061 (90.99) |  |
| Yes | 1302 (9.72) | 478 (10.71) | 422 (9.46) | 402 (9.01) |  |
| **Prior kidney failure** |  |  |  |  | 0.29 |
| No | 13165 (98.32) | 4383 (98.19) | 4399 (98.57) | 4383 (98.21) |  |
| Yes | 225 (1.68) | 81 (1.81) | 64 (1.43) | 80 (1.79) |  |
| **ACS type** |  |  |  |  | 0.06 |
| UAP | 2172 (16.22) | 695 (15.57) | 765 (17.14) | 712 (15.95) |  |
| STEMI | 7194 (53.73) | 2429 (54.41) | 2323 (52.05) | 2442 (54.72) |  |
| NSTEMI | 4024 (30.05) | 1340 (30.02) | 1375 (30.81) | 1309 (29.33) |  |
| **BMI, kg/m²** | 24.22 [22.41, 26.17] | 26.99 [25.40, 28.70] | 24.22 [23.18, 25.39] | 21.77 [20.31, 23.03] | <0.01 |
| **Total cholesterol, mg/dL** | 168.60 [140.76, 197.99] | 172.47 [143.08, 202.24] | 168.21 [140.37, 197.22] | 165.12 [139.21, 194.51] | <0.01 |
| **Triglycerides, mg/dL** | 130.20 [93.00, 189.54] | 191.31 [139.05, 266.60] | 131.08 [100.97, 172.96] | 91.23 [70.86, 120.46] | <0.01 |
| **HDL-C, mg/dL** | 39.44 [33.64, 47.18] | 35.38 [30.55, 40.99] | 39.06 [33.64, 45.63] | 44.47 [38.28, 52.98] | <0.01 |
| **LDL-C, mg/dL** | 104.15 [81.21, 129.16] | 106.34 [83.14, 130.70] | 105.57 [82.57, 130.32] | 100.54 [78.50, 125.68] | <0.01 |
| **Glucose, mmol/L** | 6.06 [5.10, 7.82] | 6.40 [5.29, 8.30] | 6.01 [5.10, 7.88] | 5.80 [4.92, 7.40] | <0.01 |
| **Isolated three-vessel**  **disease, n (%)** |  |  |  |  | 0.15 |
| No | 7481 (55.87) | 2448 (54.84) | 2494 (55.88) | 2539 (56.89) |  |
| Yes | 5909 (44.13) | 2016 (45.16) | 1969 (44.12) | 1924 (43.11) |  |

SPISE tertiles were defined as T1, 1.40 to <5.68; T2, 5.68 to <6.86; and T3, 6.86 to 15.81. Normally distributed continuous variables are shown as mean ± SD, non-normally distributed continuous variables as median (IQR), and categorical variables as n (%). SPISE, single-point insulin sensitivity estimator; T1, tertile 1; T2, tertile 2; T3, tertile 3; N, number; BMI, body mass index; HDL-C, high-density lipoprotein cholesterol; LDL-C, low-density lipoprotein cholesterol; MI, myocardial infarction; PCI, percutaneous coronary intervention; ACS, acute coronary syndrome; UAP, unstable angina pectoris; STEMI, ST-segment elevation myocardial infarction; NSTEMI, non-ST-segment elevation myocardial infarction.

**Supplementary Table 8.** Association of TyG index and TyG-BMI with left main and/or three-vessel disease.

|  | TyG index | |  | TyG-BMI | |
| --- | --- | --- | --- | --- | --- |
|  | OR (95 % CI) | *P* value |  | OR (95 % CI) | *P* value |
| TyG index, per 1-unit increase | 1.066 (1.015, 1.119) | 0.010 | TyG-BMI, per 1-unit increase | 1.001 (1.000, 1.002) | 0.057 |
| TyG index, tertiles |  |  | TyG-BMI, tertiles |  |  |
| Tertile 1 | Reference |  | Tertile 1 | Reference |  |
| Tertile 2 | 1.137 (1.053, 1.228) | 0.001 | Tertile 2 | 1.091 (1.010, 1.178) | 0.026 |
| Tertile 3 | 1.106 (1.022, 1.196) | 0.013 | Tertile 3 | 1.153 (1.066, 1.247) | <0.001 |
| *P* for trend |  | 0.017 | *P* for trend |  | <0.001 |

TyG, triglyceride-glucose index; TyG-BMI, triglyceride-glucose-body mass index; BMI, body mass index; OR, odds ratio; CI, confidence interval; MI, myocardial infarction; PCI, percutaneous coronary intervention; ACS, acute coronary syndrome.

Model: adjusted for age, sex, marital status, smoking status, history of hypertension, history of diabetes mellitus, prior MI, prior PCI, prior kidney failure, and ACS type.

**Supplementary Table 9.** Association of TyG index and TyG-BMI with isolated three-vessel disease.

|  | TyG index | |  | TyG-BMI | |
| --- | --- | --- | --- | --- | --- |
|  | OR (95 % CI) | *P* value |  | OR (95 % CI) | *P* value |
| TyG index, per 1-unit increase | 1.065 (1.009, 1.124) | 0.023 | TyG-BMI, per 1-unit increase | 1.001 (1.000, 1.002) | 0.006 |
| TyG index, tertiles |  |  | TyG-BMI, tertiles |  |  |
| Tertile 1 | Reference |  | Tertile 1 | Reference |  |
| Tertile 2 | 1.136 (1.043, 1.238) | 0.003 | Tertile 2 | 1.142 (1.049, 1.245) | 0.002 |
| Tertile 3 | 1.134 (1.038, 1.238) | 0.005 | Tertile 3 | 1.226 (1.124, 1.338) | <0.001 |
| *P* for trend |  | 0.007 | *P* for trend |  | <0.001 |

TyG, triglyceride-glucose index; TyG-BMI, triglyceride-glucose-body mass index; BMI, body mass index; OR, odds ratio; CI, confidence interval; MI, myocardial infarction; PCI, percutaneous coronary intervention; ACS, acute coronary syndrome.

Model: adjusted for age, sex, marital status, smoking status, history of hypertension, history of diabetes mellitus, prior MI, prior PCI, prior kidney failure, and ACS type.

**Supplementary Table 10.** Multiplicity-adjusted P values for interaction in subgroup analyses.

| Subgroup | P for interaction | FDR-adjusted P for interaction | Bonferroni-adjusted P for interaction |
| --- | --- | --- | --- |
| **Left main and/or three-vessel disease** | | | |
| Age | <0.001 | <0.001 | <0.001 |
| Sex | 0.399 | 0.666 | 1 |
| ACS type | 0.072 | 0.241 | 0.724 |
| Diabetes history | <0.001 | 0.004 | 0.008 |
| Hypertension history | 0.226 | 0.451 | 1 |
| Marital status | 0.172 | 0.431 | 1 |
| Smoking status | 0.876 | 0.876 | 1 |
| Prior MI | 0.787 | 0.876 | 1 |
| Prior PCI | 0.542 | 0.775 | 1 |
| Prior kidney failure | 0.812 | 0.876 | 1 |
| **Isolated three-vessel disease** | | | |
| Age | <0.001 | <0.001 | <0.001 |
| Sex | 0.842 | 0.959 | 1 |
| ACS type | 0.003 | 0.013 | 0.026 |
| Diabetes history | 0.081 | 0.27 | 0.811 |
| Hypertension history | 0.279 | 0.698 | 1 |
| Marital status | 0.557 | 0.928 | 1 |
| Smoking status | 0.889 | 0.959 | 1 |
| Prior MI | 0.475 | 0.928 | 1 |
| Prior PCI | 0.805 | 0.959 | 1 |
| Prior kidney failure | 0.959 | 0.959 | 1 |

Values are P values for interaction. Multiplicity adjustment was performed separately for each outcome using FDR and Bonferroni methods. ACS, acute coronary syndrome; FDR, false discovery rate; MI, myocardial infarction; PCI, percutaneous coronary intervention.

**Supplementary Table 11.** Association between SPISE and left main and/or three-vessel disease without exclusion of extreme SPISE values.

|  | Model 1 | |  | Model 2 | |  | Model 3 | |
| --- | --- | --- | --- | --- | --- | --- | --- | --- |
|  | OR (95 % CI) | *P* value |  | OR (95 % CI) | *P* value |  | OR (95 % CI) | *P* value |
| SPISE index, per 1-unit increase | 0.972 (0.953, 0.991) | 0.004 |  | 0.971 (0.953, 0.991) | 0.004 |  | 0.976 (0.956, 0.995) | 0.015 |
| SPISE index, tertiles |  |  |  |  |  |  |  |  |
| Tertile 1 | Reference |  |  | Reference |  |  | Reference |  |
| Tertile 2 | 0.934 (0.866, 1.006) | 0.072 |  | 0.932 (0.865, 1.005) | 0.068 |  | 0.940 (0.872, 1.014) | 0.109 |
| Tertile 3 | 0.875 (0.810, 0.944) | <0.001 |  | 0.874 (0.810, 0.943) | <0.001 |  | 0.886 (0.820, 0.957) | 0.002 |
| *P* for trend |  | <0.001 |  |  | <0.001 |  |  | 0.002 |

SPISE, single-point insulin sensitivity estimator; OR, odds ratio; CI, confidence interval; MI, myocardial infarction; PCI, percutaneous coronary intervention.

Model 1: adjusted for age, sex.
Model 2: adjusted for age, sex, marital status, and smoking status.
Model 3: adjusted for age, sex, marital status, smoking status, history of hypertension, history of diabetes mellitus, prior MI, prior PCI, prior kidney failure, ACS type.

**Supplementary Table 12.** Association between SPISE and isolated three-vessel disease without exclusion of extreme SPISE values.

|  | Model 1 | |  | Model 2 | |  | Model 3 | |
| --- | --- | --- | --- | --- | --- | --- | --- | --- |
|  | OR (95 % CI) | *P* value |  | OR (95 % CI) | *P* value |  | OR (95 % CI) | *P* value |
| SPISE index, per 1-unit increase | 0.953 (0.933, 0.975) | <0.001 |  | 0.953 (0.933, 0.975) | <0.001 |  | 0.956 (0.935, 0.978) | <0.001 |
| SPISE index, tertiles |  |  |  |  |  |  |  |  |
| Tertile 1 | Reference |  |  | Reference |  |  | Reference |  |
| Tertile 2 | 0.895 (0.823, 0.972) | 0.009 |  | 0.893 (0.822, 0.971) | 0.008 |  | 0.907 (0.834, 0.987) | 0.023 |
| Tertile 3 | 0.813 (0.747, 0.885) | <0.001 |  | 0.813 (0.747, 0.885) | <0.001 |  | 0.819 (0.752, 0.893) | <0.001 |
| *P* for trend |  | <0.001 |  |  | <0.001 |  |  | <0.001 |

SPISE, single-point insulin sensitivity estimator; OR, odds ratio; CI, confidence interval; MI, myocardial infarction; PCI, percutaneous coronary intervention.

Model 1: adjusted for age, sex.
Model 2: adjusted for age, sex, marital status, and smoking status.
Model 3: adjusted for age, sex, marital status, smoking status, history of hypertension, history of diabetes mellitus, prior MI, prior PCI, prior kidney failure, ACS type.

**Supplementary Table 13.** Association between SPISE and left main and/or three-vessel disease after exclusion of patients with cardiogenic shock or cardiac arrest.

|  | Model 1 | |  | Model 2 | |  | Model 3 | |
| --- | --- | --- | --- | --- | --- | --- | --- | --- |
|  | OR (95 % CI) | *P* value |  | OR (95 % CI) | *P* value |  | OR (95 % CI) | *P* value |
| SPISE index, per 1-unit increase | 0.973 (0.953, 0.993) | 0.01 |  | 0.973 (0.953, 0.993) | 0.01 |  | 0.978 (0.957, 0.999) | 0.037 |
| SPISE index, tertiles |  |  |  |  |  |  |  |  |
| Tertile 1 | Reference |  |  | Reference |  |  | Reference |  |
| Tertile 2 | 0.940 (0.870, 1.015) | 0.116 |  | 0.939 (0.869, 1.015) | 0.111 |  | 0.947 (0.877, 1.024) | 0.173 |
| Tertile 3 | 0.887 (0.820, 0.959) | 0.003 |  | 0.886 (0.819, 0.959) | 0.003 |  | 0.900 (0.832, 0.975) | 0.010 |
| *P* for trend |  | 0.003 |  |  | 0.003 |  |  | 0.010 |

SPISE, single-point insulin sensitivity estimator; OR, odds ratio; CI, confidence interval; MI, myocardial infarction; PCI, percutaneous coronary intervention.

Model 1: adjusted for age, sex.
Model 2: adjusted for age, sex, marital status, and smoking status.
Model 3: adjusted for age, sex, marital status, smoking status, history of hypertension, history of diabetes mellitus, prior MI, prior PCI, prior kidney failure, ACS type.

**Supplementary Table 14.** Association between SPISE and isolated three-vessel disease after exclusion of patients with cardiogenic shock or cardiac arrest.

|  | Model 1 | |  | Model 2 | |  | Model 3 | |
| --- | --- | --- | --- | --- | --- | --- | --- | --- |
|  | OR (95 % CI) | *P* value |  | OR (95 % CI) | *P* value |  | OR (95 % CI) | *P* value |
| SPISE index, per 1-unit increase | 0.954 (0.932, 0.976) | <0.001 |  | 0.954 (0.932, 0.976) | <0.001 |  | 0.957 (0.935, 0.980) | <0.001 |
| SPISE index, tertiles |  |  |  |  |  |  |  |  |
| Tertile 1 | Reference |  |  | Reference |  |  | Reference |  |
| Tertile 2 | 0.912 (0.837, 0.993) | 0.034 |  | 0.911 (0.836, 0.992) | 0.031 |  | 0.926 (0.849, 1.009) | 0.079 |
| Tertile 3 | 0.828 (0.759, 0.903) | <0.001 |  | 0.828 (0.759, 0.903) | <0.001 |  | 0.836 (0.766, 0.913) | <0.001 |
| *P* for trend |  | <0.001 |  |  | <0.001 |  |  | <0.001 |

SPISE, single-point insulin sensitivity estimator; OR, odds ratio; CI, confidence interval; MI, myocardial infarction; PCI, percutaneous coronary intervention.

Model 1: adjusted for age, sex.
Model 2: adjusted for age, sex, marital status, and smoking status.
Model 3: adjusted for age, sex, marital status, smoking status, history of hypertension, history of diabetes mellitus, prior MI, prior PCI, prior kidney failure, ACS type.

**Supplementary Table 15.** Association between SPISE and left main and/or three-vessel disease with additional adjustment for medication histories.

|  | Model 1 | |  | Model 2 | |  | Model 3 | |
| --- | --- | --- | --- | --- | --- | --- | --- | --- |
|  | OR (95 % CI) | *P* value |  | OR (95 % CI) | *P* value |  | OR (95 % CI) | *P* value |
| SPISE index, per 1-unit increase | 0.973 (0.953, 0.993) | 0.009 |  | 0.973 (0.953, 0.993) | 0.009 |  | 0.978 (0.958, 0.999) | 0.04 |
| SPISE index, tertiles |  |  |  |  |  |  |  |  |
| Tertile 1 | Reference |  |  | Reference |  |  | Reference |  |
| Tertile 2 | 0.935 (0.866, 1.009) | 0.084 |  | 0.934 (0.866, 1.008) | 0.080 |  | 0.941 (0.871, 1.016) | 0.121 |
| Tertile 3 | 0.883 (0.817, 0.955) | 0.002 |  | 0.883 (0.817, 0.954) | 0.002 |  | 0.894 (0.826, 0.968) | 0.006 |
| *P* for trend |  | 0.002 |  |  | 0.002 |  |  | 0.006 |

SPISE, single-point insulin sensitivity estimator; OR, odds ratio; CI, confidence interval; MI, myocardial infarction; PCI, percutaneous coronary intervention.

Model 1: adjusted for age, sex.
Model 2: adjusted for age, sex, marital status, and smoking status.
Model 3: adjusted for age, sex, marital status, smoking status, history of hypertension, history of diabetes mellitus, prior MI, prior PCI, prior kidney failure, ACS type, antihypertensive medication use, antidiabetic medication use, and lipid-lowering medication use.

**Supplementary Table 16.** Association between SPISE and isolated three-vessel disease with additional adjustment for medication histories.

|  | Model 1 | |  | Model 2 | |  | Model 3 | |
| --- | --- | --- | --- | --- | --- | --- | --- | --- |
|  | OR (95 % CI) | *P* value |  | OR (95 % CI) | *P* value |  | OR (95 % CI) | *P* value |
| SPISE index, per 1-unit increase | 0.954 (0.932, 0.976) | <0.001 |  | 0.954 (0.932, 0.976) | <0.001 |  | 0.957 (0.934, 0.980) | <0.001 |
| SPISE index, tertiles |  |  |  |  |  |  |  |  |
| Tertile 1 | Reference |  |  | Reference |  |  | Reference |  |
| Tertile 2 | 0.914 (0.840, 0.994) | 0.036 |  | 0.913 (0.839, 0.993) | 0.034 |  | 0.920 (0.844, 1.003) | 0.059 |
| Tertile 3 | 0.826 (0.758, 0.900) | <0.001 |  | 0.826 (0.758, 0.900) | <0.001 |  | 0.824 (0.754, 0.901) | <0.001 |
| *P* for trend |  | <0.001 |  |  | <0.001 |  |  | <0.001 |

SPISE, single-point insulin sensitivity estimator; OR, odds ratio; CI, confidence interval; MI, myocardial infarction; PCI, percutaneous coronary intervention.

Model 1: adjusted for age, sex.
Model 2: adjusted for age, sex, marital status, and smoking status.
Model 3: adjusted for age, sex, marital status, smoking status, history of hypertension, history of diabetes mellitus, prior MI, prior PCI, prior kidney failure, ACS type, antihypertensive medication use, antidiabetic medication use, and lipid-lowering medication use.

**Supplementary Table 17.** Exploratory mediation analyses of glucose and lipid markers in the association between SPISE and left main and/or three-vessel disease.

| Outcome | Candidate metabolic marker | Estimated indirect effect / ACME | 95% CI | P value | Estimated direct effect / ADE | Total effect | Estimated proportion |
| --- | --- | --- | --- | --- | --- | --- | --- |
| Left main and/or three-vessel disease | Glucose | -0.00082 | (-0.00129, -0.00039) | <0.001 | -0.00482 | -0.00563 | 14.50% |
| Left main and/or three-vessel disease | Total cholesterol | -0.00042 | (-0.00069, -0.00019) | <0.001 | -0.00521 | -0.00562 | 7.40% |
| Left main and/or three-vessel disease | LDL-C | -0.00049 | (-0.00085, -0.00018) | 0.001 | -0.00513 | -0.00563 | 8.80% |
| Isolated three-vessel disease | Glucose | -0.00072 | (-0.00124, -0.00019) | 0.006 | -0.00979 | -0.01051 | 6.80% |
| Isolated three-vessel disease | Total cholesterol | -0.00041 | (-0.00073, -0.00016) | <0.001 | -0.0101 | -0.01051 | 3.90% |
| Isolated three-vessel disease | LDL-C | -0.00043 | (-0.00080, -0.00009) | 0.012 | -0.01008 | -0.01052 | 4.10% |

ACME and ADE are reported as standard outputs from the mediation model. Given the cross-sectional design, these estimates should be interpreted as exploratory findings rather than evidence of causal mediation.

**Supplementary Table 18.** The names of principal investigators and participating centers.

| **ID** | **Hospitals** | **Territories** | **Provinces** | **City** | **Investigator(s)** |
| --- | --- | --- | --- | --- | --- |
| 1 | Shanxi Cardiovascular Hospital | Northern China | Shanxi | Taiyuan | Bao Li |
| 2 | Nanjing Drum Tower Hospital, The Affiliated Hospital of Nanjing University Medical School | Eastern China | Jiangsu | Nanjing | Biao Xu, Guangshu Han |
| 3 | Hainan General Hospital | Southern China | Hainan | Haikou | Bin Li |
| 4 | The Second Hospital of Jilin University | Northeast China | Jilin | Changchun | Bin Liu |
| 5 | The 2nd Affiliated Hospital of Harbin Medical University | Northeast China | Heilongjiang | Harbin | Bo Yu |
| 6 | The Ninth Hospital Affiliated to Shanghai Jiaotong University School of Medicine | Eastern China | Shanghai | Shanghai | Changqian Wang |
| 7 | Henan Provincial People's Hospital | Central China | Henan | Zhengzhou | Chuanyu Gao |
| 8 | Shanxi Provincial People's Hospital | Northern China | Shanxi | Taiyuan | Chunlin Lai |
| 9 | Xinqiao Hospital, Third Military Medical University | Southwest China | Chongqing | Chongqing | Cui Bin, Lan Huang |
| 10 | China Meitan General Hospital | Northern China | Beijing | Beijing | Di Wu |
| 11 | The 309th Hospital of Chinese People's Liberation Army | Northern China | Beijing | Beijing | Fakuan Tang, Jun Xiao |
| 12 | Zhongda Hospital, Southeast University | Eastern China | Jiangsu | Nanjing | Genshan Ma |
| 13 | The First Affiliated Hospital of Liaoning Medical University | Northeast China | Liaoning | Jinzhou | Guizhou Tao |
| 14 | Xinjiang Uygur Autonomous Region People’s Hospital | Northwest China | Xinjiang | Urumchi | Guoqing Li |
| 15 | Sir Run Run Shaw Hospital, College of Medicine, Zhejiang | Eastern China | Zhejiang | Hangzhou | Guosheng Fu |
| 16 | Beijing Friendship Hospital, Capital Medical University | Northern China | Beijing | Beijing | Hongwei Li |
| 17 | The First Affiliated Hospital of Bengbu Medical College | Eastern China | Anhui | Bengbu | Honhju Wang |
| 18 | General Hospital of TISCO | Northern China | Shanxi | Taiyuan | Huifeng Wang |
| 19 | Dongguan People's Hospital | Southern China | Guangdong | Dongguan | Jianfeng Ye |
| 20 | Panyu Hospital of Chinese Medicine | Southern China | Guangdong | Guangzhou | Jianhao Li |
| 21 | Peking University First Hospital | Northern China | Beijing | Beijing | Jie Jiang |
| 22 | Sun Yat-sen Memorial Hospital, Sun Yat-sen University | Southern China | Guangdong | Guangzhou | Jingfeng Wang |
| 23 | Guangdong General Hospital | Southern China | Guangdong | Guangzhou | Jiyan Chen |
| 24 | Hospital of Xinjiang Production & Construction Corps | Northwest China | Xinjiang | Urumchi | Junming Liu |
| 25 | The Military General Hospital of Beijing PLA | Northern China | Beijing | Beijing | Junxia Li |
| 26 | The First Affiliated Hospital of Guangxi Medical University | Southern China | Guangxi | Nanning | Lang Li |
| 27 | Tongren Hospital Affiliated to Shanghai Jiaotong University School of Medicine | Eastern China | Shanghai | Shanghai | Li Jiang |
| 28 | Binzou City Center Hospital | Eastern China | Shandong | Binzhou | Lijun Meng |
| 29 | The First Affiliated Hospital of Zhengzhou University | Central China | Henan | Zhengzhou | Ling Li |
| 30 | Xijing Hospital | Northwest China | Shaanxi | Xi'an | Ling Tao |
| 31 | The Affiliated Hospital of Guizhou Medical University | Southwest China | Guizhou | Guiyang | Lirong Wu |
| 32 | First Affiliated Hospital of the People's Liberation Army General Hospital | Northern China | Beijing | Beijing | Miao Tian |
| 33 | The Second People's Hospital of Yunnan Province | Southwest China | Yunnan | Kunming | Minghua Han |
| 34 | Haikou People's Hospital | Southern China | Hainan | Haikou | Moshui Chen |
| 35 | Gansu Provincial Hospital | Northwest China | Gansu | Lanzhou | Ping Xie |
| 36 | The First Affiliated Hospital of Henan University of Science and Technology | Central China | Henan | Luoyang | Pingshuan Dong |
| 37 | Chenzhou First People's Hospital | Central China | Hunan | Chenzhou | Qiaoqing Zhong |
| 38 | People’s Hospital of Qinghai Province | Northwest China | Qinghai | Xining | Rong Chang |
| 39 | Affiliated Hospital of Ningxia Medical University | Northwest China | Ningxia | Yinchuan | Shaobin Jia |
| 40 | Beijing Anzhen Hospital, Capital Medical University | Northern China | Beijing | Beijing | Shaoping Nie, Xiaohui Liu |
| 41 | North Jiangsu People's Hospital | Eastern China | Jiangsu | Yangzhou | Shenghu He |
| 42 | Shanghai Sixth People's Hospital | Eastern China | Shanghai | Shanghai | Shixin Ma |
| 43 | The First Hospital of Handan | Northern China | Hebei | Handan | Shuanli Xin |
| 44 | Huai'an First People's Hospital | Eastern China | Jiangsu | Huai'an | Shuren Ma |
| 45 | The First Affiliated Hospital of Chongqing Medical University | Southwest China | Chongqing | Chongqing | Suxin Luo |
| 46 | Navy General Hospital | Northern China | Beijing | Beijing | Tianchang Li |
| 47 | Zhejiang Provincial Hospital of TCM | Eastern China | Zhejiang | Hangzhou | Wei Mao |
| 48 | The Third Xiangya Hospital of Central South University | Central China | Hunan | Changsha | Weihong Jiang |
| 49 | Affiliated Hospital of Qinghai University | Northwest China | Qinghai | Xining | Weijun Liu |
| 50 | Teda International Cardiovascular Hospital | Northern China | Tianjin | Tianjin | Wenhua Lin |
| 51 | The Second Hospital of Hebei Medical University | Northern China | Hebei | Shijiazhuang | Xianghua Fu |
| 52 | Changhai Hospital of Shanghai | Eastern China | Shanghai | Shanghai | Xianxian Zhao |
| 53 | The Second Affiliated Hospital to Nanchang University | Eastern China | Jiangxi | Nanchang | Xiaoshu Cheng |
| 54 | Hebei General Hospital | Northern China | Hebei | Shijiazhuang | Xiaoyong Qi |
| 55 | Inner Mongolia People's Hospital | Northern China | Inner Mongolia | Hohhot | Xingsheng Zhao |
| 56 | The General Hospital of Shenyang Military Region | Northeast China | Liaoning | Shenyang | Yaling Han |
| 57 | The First Hospital of Jilin University | Northeast China | Jilin | Changchun | Yang Zheng |
| 58 | Tianjin Chest Hospital | Northern China | Tianjin | Tianjin | Yin Liu |
| 59 | Hunan Provincial People's Hospital | Central China | Hunan | Changsha | Ying Guo |
| 60 | People's Hospital of Yuxi City | Southwest China | Yunnan | Yuxi | Yinglu Hao |
| 61 | The People's Hospital of Guangxi Zhuang Autonomous Region | Southern China | Guangxi | Nanning | Yingzhong Lin |
| 62 | The First Teaching Hospital of Xinjiang Medical University | Northwest China | Xinjiang | Urumchi | Yitong Ma |
| 63 | Baogang Hospital | Northern China | Inner Mongolia | Baotou | Yongdong Li |
| 64 | Tianjin Medical University General Hospital | Northern China | Tianjin | Tianjin | Yuemin Sun |
| 65 | The Second Affiliated Hospital of Zhengzhou University | Central China | Henan | Zhengzhou | Yulan Zhao |
| 66 | Nanfang Hospital of Southern Medical University | Southern China | Guangdong | Guangzhou | Yuqing Hou |
| 67 | The First Affiliated Hospital to Nanchang University | Eastern China | Jiangxi | Nanchang | Zeqi Zheng |
| 68 | The First Affiliated Hospital of Lanzhou University | Northwest China | Gansu | Lanzhou | Zheng Zhang |
| 69 | The Third Hospital of Shijiazhuang | Northern China | Hebei | Shijiazhuang | Zhenguo Ji |
| 70 | Wuxi People's Hospital | Eastern China | Jiangsu | Wuxi | Zhenyu Yang |
| 71 | Jiangsu Province Hospital | Eastern China | Jiangsu | Nanjing | Zhijian Yang |
| 72 | The Second Hospital of Shanxi Medical University | Northern China | Shanxi | Taiyuan | Zhiming Yang |
| 73 | The Affiliated Hospital of Xuzhou Medical College | Eastern China | Jiangsu | Xuzhou | Zhirong Wang |
| 74 | Southwest Hospital, Third Military Medical University | Southwest China | Chongqing | Chongqing | Zhiyuan Song |
| 75 | The First Affiliated Hospital of Xi’an Jiaotong University | Northwest China | Shaanxi | Xi'an | Zuyi Yuan |
| 76 | Yangzhou First People's Hospital | Eastern China | Jiangsu | Yangzhou | Aihua Li |
| 77 | Hospital 463 of Chinese People's Liberation Army | Northeast China | Liaoning | Shenyang | Bosong Yang |
| 78 | The Central Hospital of Mianyang | Northwest China | Sichuan | Mianyang | Caidong Luo |
| 79 | Liaocheng People's Hospital | Eastern China | Shandong | Liaocheng | Chunyan Zhang |
| 80 | Yancheng Third People's Hospital | Eastern China | Jiangsu | Yancheng | Chunyang Wu |
| 81 | The Second Xiangya Hospital of Central South University | Central China | Hunan | Changsha | Daoquan Peng |
| 82 | The Central Hospital of Panzhihua | Northwest China | Sichuan | Panzhihua | Dawen Xu |
| 83 | The First Hospital of Qiqihaer City | Northeast China | Heilongjiang | Qiqihaer | Gang Xu |
| 84 | The Third the People‘s Hospital of Bengbu | Eastern China | Anhui | Bengbu | Gengsheng Sang |
| 85 | The First Hospital of Jiamusi | Northeast China | Heilongjiang | Jiamusi | Guixia Zhang |
| 86 | Zhoushan People's Hospital | Eastern China | Zhejiang | Zhoushan | Guoxiong Chen |
| 87 | Dalian Municipal Central Hospital | Northeast China | Liaoning | Dalian | Hailong Lin |
| 88 | Renmin Hospital of Wuhan University | Central China | Hubei | Wuhan | Hong Jiang |
| 89 | Ningxia People's Hospital | Northwest China | Ningxia | Yinchuan | Hong Luan |
| 90 | The First People's Hospital of Yunnan Province (Kunhua Hospital) | Northwest China | Yunnan | Kunming | Hong Zhang |
| 91 | The Central Hospital of Zhoukou | Central China | Henan | Zhoukou | Hualing Liu |
| 92 | Anyang District Hospital | Central China | Henan | Anyang | Hui Liu |
| 93 | Sichuan Provincial People’s Hospital | Northwest China | Sichuan | Chengdu | Jianhong Tao |
| 94 | Mudanjiang Cardiovascular Disease Hospital | Northeast China | Heilongjiang | Mudanjiang | Jianwen Liu |
| 95 | Yichang Central Hospital | Central China | Hubei | Yichang | Jiawang Ding |
| 96 | Qilu Hospital of Shandong University | Eastern China | Shandong | Jinan | Jifu Li |
| 97 | Affiliated Hospital of Jiangsu University | Eastern China | Jiangsu | Zhenjiang | Jinchuan Yan |
| 98 | The First People's Hospital of Nanning City | Southern China | Guangxi | Nanning | Jinru Wei |
| 99 | The First Affiliated Hospital of Fujian Medical University | Eastern China | Fujian | Fuzhou | Jinzi Su |
| 100 | Chengdu Third People’s Hospital | Northwest China | Sichuan | Chengdu | Jiong Tang |
| 101 | Yantaishan hospital | Eastern China | Shandong | Yantai | Juexin Fan |
| 102 | Qingdao Municipal Hospital | Eastern China | Shandong | Qingdao | Jun Guan |
| 103 | Zhongshan Hospital Affiliated to Fudan University | Eastern China | Shanghai | Shanghai | Junbo Ge |
| 104 | Longyan First Hospital | Eastern China | Fujian | Longyan | Kaihong Chen |
| 105 | Affiliated Hospital of Guangdong Medical College | Southern China | Guangdong | Guangzhou | Keng Wu |
| 106 | Jiangxi Provincial People's Hospital | Eastern China | Jiangxi | Nanchang | Lang Ji |
| 107 | Anhui Provincial Hospital | Eastern China | Anhui | Hefei | Likun Ma |
| 108 | Xiangtan City Central Hospital | Central China | Hunan | Xiangtan | Lilong Tang |
| 109 | The First Hospital of Haerbin City | Northeast China | Heilongjiang | Harbin | Lin Wei |
| 110 | Central Hospital Affiliated to Shenyang Medical College | Northeast China | Liaoning | Shenyang | Man Zhang, Kaiming Chen |
| 111 | The Central Hospital of Wuhan | Central China | Hubei | Wuhan | Manhua Chen |
| 112 | Hangzhou First People's Hospital | Eastern China | Zhejiang | Hangzhou | Ningfu Wang |
| 113 | The Central Hospital of Xuzhou | Eastern China | Jiangsu | Xuzhou | Peiying Zhang |
| 114 | The Second hospital of Dalian Medical University | Northeast China | Liaoning | Dalian | Peng Qu |
| 115 | The First Affiliated Hospital of Liaoning University of Traditional Chinese Medicine | Northeast China | Liaoning | Shenyang | Ping Hou |
| 116 | Beijing Tsinghua Changgung Hospital | Northern China | Beijing | Beijing | Ping Zhang |
| 117 | Guizhou Provincial People's Hospital | Northwest China | Guizhou | Guiyang | Qiang Wu |
| 118 | The First Affiliated Hospital of Xiamen University | Eastern China | Fujian | Xiamen | Qiang Xie |
| 119 | Quanzhou First Hospital | Eastern China | Fujian | Quanzhou | Rong Lin |
| 120 | Wuzhou People's Hospital | Southern China | Guangxi | Wuzhou | Shaowu Ye |
| 121 | The Central Hospital of Jilin | Northeast China | Jilin | Changchun | Shuangbin Li |
| 122 | Xiangya Hospital Central South University | Central China | Hunan | Changsha | Tianlun Yang |
| 123 | Guangzhou Red Cross Hospital | Southern China | Guangdong | Guangzhou | Tongguo Wu |
| 124 | The First Affiliated Hospital of Guangzhou Medical College | Southern China | Guangdong | Guangzhou | Wei Wang |
| 125 | The First Affiliated Hospital of Wenzhou Medical University | Eastern China | Zhejiang | Wenzhou | Weijian Huang |
| 126 | The Second Affiliated Hospital of Soochow University | Eastern China | Jiangsu | Suzhou | Weiting Xu |
| 127 | Wuhan Asia Heart Hospital | Central China | Hubei | Wuhan | Xi Su |
| 128 | The First Affiliated Hospital of Soochow University | Eastern China | Jiangsu | Suzhou | Xiangjun Yang |
| 129 | Affiliated Hospital of Yan'an University | Northwest China | Shaanxi | Yan'an | Xiaochuan Ma |
| 130 | The First People's Hospital of Jining | Eastern China | Shandong | Jining | Xiaofei Sun |
| 131 | The Central Hospital of Taiyuan | Northern China | Shanxi | Taiyuan | Xiaoping Chen |
| 132 | West China Hospital of Sichuan University | Northwest China | Sichuan | Chengdu | Xiaoping Chen |
| 133 | The Third Affiliated Hospital of Guangzhou Medical College | Southern China | Guangdong | Guangzhou | Ximing Chen |
| 134 | The First Affiliated Hospital of Wannan Medical College | Eastern China | Anhui | Wuhu | Xingsheng Tang |
| 135 | Tangdu Hospital of The Fourth Military Medical University | Northwest China | Shaanxi | Xi'an | Xue Li |
| 136 | Shanghai East Hospital Affiliated to Tongji University | Eastern China | Shanghai | Shanghai | Xuebo Liu |
| 137 | Xiamen Cardiovascular Disease Hospital | Eastern China | Fujian | Xiamen | Yan Wang |
| 138 | Zhongnan hospital of Wuhan University | Central China | Hubei | Wuhan | Yanggan Wang |
| 139 | Fujian Provincial Hospital | Eastern China | Fujian | Fuzhou | Yansong Guo |
| 140 | The First Affiliated hospital of Dalian Medical University | Northeast China | Liaoning | Dalian | Yanzong Yang |
| 141 | The First People's Hospital of Changde | Central China | Hunan | Changde | Yi Huang |
| 142 | The First Affiliated Hospital of China Medical University | Northeast China | Liaoning | Shenyang | Yingxian Sun |
| 143 | The Fourth Affiliated Hospital of China Medical University | Northeast China | Liaoning | Shenyang | Yuanzhe Jin |
| 144 | Cangzhou Central Hospital | Northern China | Hebei | Cangzhou | Zesheng Xu |
| 145 | The Central Hospital of Shaoyang | Central China | Hunan | Shaoyang | Zewei Ouyang |
| 146 | The People's Hospital of Liaoning Province | Northeast China | Liaoning | Shenyang | Zhanquan Li |
| 147 | The First Affiliated Hospital of Jiamusi University | Northeast China | Heilongjiang | Jiamusi | Zhaofa He |
| 148 | Tangshan Gongren Hospital | Northern China | Hebei | Tangshan | Zheng Ji |
| 149 | Huaibei Miners General Hospital | Eastern China | Anhui | Huaibei | Zhenqi Su |
| 150 | Linyi People's Hospital | Eastern China | Shandong | Linyi | Zhihong Ou |
| 151 | Chongqing Hechuan District People's Hospital | Southwest China | Chongqing | Chongqing | Xin Tang |
| 152 | Yuzhou City Central Hospital | Central China | Henan | Xuchang | Qinfeng Su |
| 153 | Jianshui County People's Hospital | Southwest China | Yunnan | Honghe | Weiqing Fan |
| 154 | Dunhua City Hospital | Northeast China | Jilin | Dunhua | Fanju Meng |
| 155 | Shenyang City Electricity Central Hospital | Northeast China | Liaoning | Shenyang | Jing Xu |
| 156 | Shanghai Jingan District Shibei Hospital | Eastern China | Shanghai | Shanghai | Bin Wang |
| 157 | Beijing Fangshan District First Hospital | Northern China | Beijing | Beijing | Xuemei Peng |
| 158 | Hebei Daming County People's Hospital | Northern China | Hebei | Handan | Haiping Guo |
| 159 | Jiangsu Binhai County People's Hospital | Eastern China | jiangsu | Yancheng | Yonglin Zhang |
| 160 | The First People's Hospital of Longquanyi District,Chengdu | Southwest China | Sichuan | Chengdu | Wei Tuo |
| 161 | Guangxi Hengxian County People's Hospital | Southern China | Guangxi | Nanning | Xianan Zhang |
| 162 | Hunan Changsha County First People's Hospital | Central China | Hunan | Changsha | Siding Wang |
| 163 | People's Hospital of Wugang | Central China | Hunan | Shaoyang | JiaoMei Yang |
| 164 | Longhui County People's Hospital | Central China | Hunan | Shaoyang | Xiaojun Wang |
| 165 | Heilongjiang Fujin City Central Hospital | Northeast China | Heilongjiang | Jiamusi | Jiyan Yin |
| 166 | Dalian Fourth People's Hospital | Northeast China | Liaoning | Dalian | Huifang Zhang |
| 167 | People's Hospital of Haixi Autonomous Prefecture | Northwest China | Qianghai | Haixi | Wantai Li |
| 168 | General Hospital of Guangzhou Military Command | Southern China | Guangdong | Guangzhou | Yanlie Zheng |
| 169 | The First People's Hospital of Horqin District, Tongliao City | Northern China | Inner Mongolia | Tongliao | Junping Fang |
| 170 | Guiyang Sixth People 's Hospital | Southwest China | Guizhou | Guiyang | Kalan Luo |
| 171 | Geological Mining Hospital of Hunan Province | Central China | Hunan | Changsha | Naiyi Liang |
| 172 | Zhangzhou Municipal Hospital of Fujian Province | Eastern China | Fujian | Zhangzhou | Changyong Liu |
| 173 | Jining City Yanzhou District People's Hospital | Eastern China | Shandong | Jining | Jian Yang |
| 174 | The People's Hospital Feixian | Eastern China | Shandong | Linyi | Honghua Deng |
| 175 | Tangshan City Fengrun District People 's Hospital | Northern China | Hebei | Tangshan | Lin Wang |
| 176 | Qian'an People 's Hospital | Northern China | Hebei | Tangshan | Yuheng Yang |
| 177 | Yuzhong County People's Hospital | Northwest China | Gansu | Lanzhou | Xiaowei Peng |
| 178 | Baiyin Cite Center Hospital | Northwest China | Gansu | Baiyin | Fang Zhao |
| 179 | Mingguang People's Hospital | Eastern China | Anhui | Chuzhou | Yong Li |
| 180 | Xihua County People's Hospital | Central China | Henan | Zhoukou | Chuntong Wang |
| 181 | Zhalantun People's Hospital | Northern China | Inner Mongolia | Hulunbeier | Yuhua Zhu |
| 182 | Fengrun District Second People's Hospital | Northern China | Hebei | Tangshan | Jingshan Zhao |
| 183 | Zhangping City Hospital | Eastern China | Fujian | Zhangpin | Jinxing Yi |
| 184 | Fuqing Cite Hospital | Eastern China | Fujian | Fuqing | Ping Chen |
| 185 | The Eight Affiliated Hospital, Sun Yat-sen University | Southern China | Guangdong | Guangzhou | Nan Jia |
| 186 | The First Affiliated Hospital of Qiqihar Medical Hospital | Northeast China | Heilongjiang | Qiqihaer | Yanli Wang |
| 187 | Wuhan University of Science and Technology Hospital | Central China | Hubei | Wuhan | Jing Hu |
| 188 | Baotou City Center Hospital | Northern China | Inner Mongolia | Baotou | Ruiping Zhao |
| 189 | Shanghai Jiading District Center Hospital | Eastern China | Shanghai | Shanghai | Xia Chen |
| 190 | Shanghai Jiangwan Hospital | Eastern China | Shanghai | Shanghai | Aiping Li |
| 191 | Datong City Second People's Hospital | Northern China | Shanxi | Datong | Xiaoqin Zhang |
| 192 | Shouyang County People's Hospital | Northern China | Shanxi | Jinzhong | Buqiang Zhang |
